# From Media Attention to Corrective Action: Extending the IPMI Model with a Multigroup Comparison by Media Literacy

**DOI:** 10.3390/bs16030361

**Published:** 2026-03-04

**Authors:** Zhiqi Wang, Luis Fernando Morales Morante

**Affiliations:** Departament of Advertising, Public Relations and Audiovisual Communication, Autonomous University of Barcelona, 08193 Bellaterra, Spain; zhiqi.wang2@autonoma.cat

**Keywords:** food health misinformation, Influence of Presumed Media Influence (IPMI), personal norms, corrective behavior, media literacy

## Abstract

Food health misinformation poses risks to public well-being, often spreading through social media and interpersonal contexts. This study extends the Influence of Presumed Media Influence (IPMI) model to explain how individuals move from attention to misinformation toward corrective behavioral intentions, while examining the moderating role of media literacy. Data were collected from a national online survey of 1021 Chinese adults, measuring media attention, presumed exposure of others, perceived negative influence, personal norms, media literacy, and correction intentions. Structural equation modeling supported a positive serial mediation chain, in which media attention was positively associated with presumed exposure of others, which in turn positively predicted presumed negative influence on others, leading to stronger personal norms and, ultimately, greater corrective behavioral intentions. Multi-group analysis showed that media literacy moderated this process: lower literacy amplified the link from perceived influence to norms, while higher literacy strengthened the link from norms to behavior. These findings advance the IPMI framework by highlighting media literacy as a critical boundary condition and suggest that interventions should not only correct misinformation but also foster responsibility for others and enhance media literacy to encourage user-driven corrections.

## 1. Introduction

In today’s interconnected media landscape, food and health-related misinformation, ranging from exaggerated risks of additives to unverified dietary benefits, spreads rapidly across diverse channels, often without scientific validation. Such content can distort dietary decisions, fuel public confusion, and erode trust in health guidance (Lewandowsky et al., 2017; Pennycook & Rand, 2019). In contexts highly sensitive to food safety, such as China, these effects are especially pronounced (Guess et al., 2019). While prior research has examined misinformation correction strategies by highlighting the roles of timing, source credibility, and message framing (Tandoc et al., 2018), less is known about the psychological drivers that prompt individuals to proactively correct false information. This gap is critical because in participatory media environments ordinary users play a growing role in detecting and challenging misinformation.

The Influence of Presumed Media Influence (IPMI) model provides a powerful framework grounded in psychological mechanisms for understanding such behaviors. Unlike traditional media effects models, which emphasize direct effects on the self, IPMI posits that perceptions of media effects on others can shape individuals’ own attitudes and behaviors (Gunther & Storey, 2003). This process reflects a perception-driven psychological chain: attention to misinformation leads individuals to presume others’ exposure, evaluate its potential harm, activate personal norms (felt obligations to act), and ultimately engage in corrective behavior. In the misinformation context, individuals may not personally believe false claims but may still feel compelled to act in order to protect others, particularly when content is emotionally charged or socially consequential (Ho et al., 2015; M. Y. Wang & Huang, 2020). From a psychological perspective, IPMI thus illustrates how perception and normative processes guide prosocial engagement with misinformation.

Yet important gaps remain. Few studies have traced the full psychological pathway from media attention to corrective behavior, especially in the context of food-related misinformation, which often appears benign, circulates within personal networks, and resists debunking (Berinsky, 2017). Moreover, most IPMI research treats audiences as uniform, overlooking differences in media literacy, which represents a core psychological capacity to access, evaluate, and respond to media content (Livingstone, 2004). Individuals with higher literacy may critically evaluate information, recognize misinformation, and translate personal norms into action, whereas those with lower literacy may perceive harm but feel powerless to respond (Vraga & Tully, 2021). These individual differences highlight the importance of integrating media literacy into models of misinformation correction.

This study addresses these gaps by testing an integrative model that links attention to food health misinformation with corrective behavior through presumed exposure, perceived harm, and personal norms, while incorporating media literacy as a central boundary condition. In extending the IPMI model, this study highlights media literacy as a critical factor that shapes not only how individuals perceive misinformation’s influence but also how personal norms are translated into corrective actions. By positioning media literacy at the center of the framework, this study contributes a novel psychological dimension to the IPMI model, bridging perception, cognitive evaluation, and normative activation. This framework clarifies both the general psychological mechanisms and the individual differences that drive corrective engagement, thereby advancing theoretical understanding of misinformation correction and offering practical insights for designing psychologically informed interventions.

### 1.1. Corrective Behavior in the Context of Food Health Misinformation

Food health misinformation has emerged as a growing concern in both research and policy. Unlike political misinformation, which often triggers ideological defenses or partisan polarization, food health misinformation usually appears as seemingly benign advice grounded in wellness beliefs or traditional knowledge. Such claims, including exaggerated risks of food additives or pseudoscientific dietary recommendations, can confuse the public, undermine trust in health authorities, and lead to suboptimal or even harmful health behaviors (Lewandowsky et al., 2017; Pennycook & Rand, 2019). In countries with a history of food safety controversies, such as China, these risks are amplified by heightened public sensitivity to food-related issues (Guess et al., 2019). This makes food health misinformation a distinct phenomenon with strong interpersonal and psychological implications.

Early studies of corrective behavior largely focused on message-level features. Research identified conditions under which corrections are more persuasive, such as timing, source credibility, message framing, narrative structure, authority cues, and emotional appeals. For instance, corrections delivered shortly after misinformation exposure and from credible sources are more effective in reducing belief, while neutral, fact-based messages often outperform sarcastic or combative approaches (Pennycook & Rand, 2019; Tandoc et al., 2018). Yet, these studies prioritize message design and largely overlook the role of individual psychological motivations in driving corrective actions.

Subsequent research shifted attention to cognitive and affective mechanisms. Findings on belief perseverance, confirmation bias, and emotional resistance illustrate why misinformation can continue to influence attitudes even after correction, a phenomenon known as the “continued influence effect” (Lewandowsky et al., 2017). These insights demonstrate that cognitive and emotional processes play a crucial role in shaping responses to correction efforts. However, little is known about how such processes interact with broader normative pressures or social contexts to influence user-driven correction.

More recently, scholars have examined social and normative factors that motivate corrective behavior, particularly in participatory media environments. Correction intentions are influenced by perceived social norms, identity-related motivations, and personal obligations. For example, individuals are more likely to challenge misinformation when corrective actions align with community expectations or reinforce their normative identities (Vraga & Bode, 2017; Margolin et al., 2018). These studies highlight the importance of contextual and relational factors, but they rarely explain how personal norms are activated in misinformation contexts or how they interact with cognitive and affective processes.

Finally, research has begun to adopt audience-centered perspectives, shifting from platform-driven solutions to user-initiated responses. This stream emphasizes user agency by asking why individuals choose to correct misinformation, rather than only how corrections work. Despite these advances, existing studies remain fragmented, often treating message-level features, cognitive–emotional mechanisms, and normative drivers in isolation. Moreover, food health misinformation remains underexplored, despite its unique interpersonal salience and potential to activate responsibility for others.

Taken together, these gaps point to the necessity of an integrative framework that explains corrective behavior as a sequential process linking attention, perceptions of others’ exposure, perceived harm, and personal norms to corrective action. The present study addresses this need by drawing on the Influence of Presumed Media Influence (IPMI) model, which provides a process-oriented lens for examining how psychological and normative mechanisms jointly motivate user-driven correction in the context of food health misinformation.

### 1.2. The Influence of Presumed Media Influence (IPMI) as a Theoretical Framework

Building on prior literature that examined message-level features, cognitive–emotional processes, and social norms largely in isolation, there remains a need for a psychological framework that integrates these perspectives to explain user-driven corrective behavior. To address this, the present study draws on the Influence of Presumed Media Influence (IPMI) model (Gunther & Storey, 2003). Unlike conventional media effects models that focus on how media shape individuals’ own attitudes or behaviors, IPMI emphasizes that people are also influenced by their perception of how media content affects others. These perceptions trigger psychological processes that are rooted not in self-concern but in concern for others’ well-being.

The IPMI framework is particularly well-suited to the study of health misinformation. Individuals may not personally believe misleading claims, but they can still perceive such content as socially harmful. Prior applications of IPMI have shown that presumed influence on others can predict meaningful outcomes, such as support for regulation or information-sharing intentions, across diverse contexts including political disinformation (Tsfati & Cappella, 2005), environmental campaigns (Ho et al., 2015), and public health communication (M. Y. Wang & Huang, 2020). Importantly, IPMI illustrates a perception-driven psychological pathway: attention to misinformation fosters perceptions of others’ exposure, which heighten evaluations of potential harm, activate personal norms (felt obligations to act), and ultimately motivate corrective behavior. In this way, IPMI provides an integrative mechanism linking perception, cognition, and normative activation to prosocial engagement.

Food health misinformation represents a context where this pathway is especially salient due to its interpersonal nature. Unlike political opinions, which often divide along ideological lines, food health claims are frequently shared within family or caregiving networks. This interpersonal proximity increases concern for how others may be affected and strengthens the likelihood of norm activation. Thus, food health misinformation provides a unique testing ground for IPMI by highlighting how perceptions of harm to close others can translate into corrective actions.

Although IPMI conceptually builds upon the third-person effect (TPE), it goes beyond simple perceptual asymmetries. Whereas TPE research emphasizes attitudinal responses such as support for censorship, IPMI explicates a fuller motivational chain, showing how perceived media influence on others can be transformed into corrective behavior through personal norms. This distinction underscores IPMI’s value as a psychological model that links concern for others to proactive action.

Despite its theoretical utility, IPMI has rarely been operationalized as a sequential behavioral process in the domain of health misinformation. Existing studies often test partial relationships, such as the link between media exposure and concern for others, without modeling the entire motivational pathway. The present study addresses this gap by adopting IPMI as a full-process framework and applying it to a novel and socially significant domain: food health misinformation.

### 1.3. From Self’s Attention to Presumed Exposure on Others

The Influence of Presumed Media Influence (IPMI) model suggests that individuals’ perceptions of how media affect others can shape their own attitudes and behaviors (Gunther & Storey, 2003). When individuals themselves are exposed to misinformation, such exposure makes the content more cognitively accessible and salient, leading them to project this experience onto others. This projection is consistent with the availability heuristic, whereby people estimate how widely an event occurs based on how easily it comes to mind (Tversky & Kahneman, 1973).

In the case of food health misinformation, the proliferation of digital platforms amplifies this tendency. Because sharing is effortless and content spreads virally, individuals often assume that if they have encountered a piece of misinformation, many others are also likely to have done so. This process links self-attention with presumed exposure on others, setting the stage for further evaluations of harm.

**H1.**  *Individuals’ attention to food health misinformation is positively associated with their presumed exposure to such misinformation on others.*

### 1.4. From Presumed Exposure on Others to Perceived Negative Influence on Others

Within the IPMI framework, perceiving that others pay greater attention to misinformation often leads individuals to infer that such attention will have harmful consequences. When people believe that others devote more attention to food health misinformation, they are more likely to assume that these false claims will be processed, remembered, and eventually acted upon. This reasoning is supported by the idea that sustained attention increases the likelihood of internalizing information and integrating it into decision-making (Lang, 2000).

In this way, presumed exposure on others functions as a cognitive signal of anticipated harm: the more attention others are perceived to give to misinformation, the stronger the expectation of its negative influence. Prior studies have shown that higher presumed attention to misleading content predicts stronger inferences of harmful media effects on others (Tsfati & Cohen, 2005).

**H2.**  *Perceived attention to food health misinformation by others is positively associated with individuals’ perception of its negative influence on others.*

### 1.5. From Perceived Negative Influence on Others to Personal Norms

When individuals recognize that misinformation may cause harm to others, this perception can activate personal norms, fostering a sense of moral responsibility to intervene. According to the Norm Activation Model (NAM), awareness of consequences and ascription of responsibility are critical precursors for the internalization of personal norms (Schwartz, 1977). Unlike general attitudes, personal norms reflect an internalized obligation that guides behavior when one perceives that others may suffer negative consequences.

This mechanism has been well-documented in various domains. For instance, in environmental behavior, individuals who recognize ecological harm and feel personally responsible are more likely to act pro-environmentally (Bamberg & Möser, 2007). A similar process can be expected in the context of food health misinformation. When people perceive that misinformation misleads others and threatens their well-being, they are likely to experience an internalized responsibility to protect others from such harm. This sense of obligation is reflected in the activation of personal norms that motivate corrective engagement.

**H3.**  *Perceived negative influence of food health misinformation on others is positively associated with individuals’ activation of personal norms to engage in correction behavior.*

### 1.6. From Personal Norms to Corrective Behavioral Outcomes

Once personal norms are activated, they can directly shape individuals’ intentions to engage in corrective actions. According to the Norm Activation Model (NAM) and related value–belief–norm theories, personal norms represent an internalized sense of obligation that translates perceptions of harm into behavioral intentions (Schwartz, 1977). In the context of misinformation correction, this means that when people feel a normative responsibility to protect others from harm, they are more inclined to consider taking corrective actions.

Empirical evidence supports this linkage. For example, Bode and Vraga (2015) showed that individuals who felt a personal responsibility to intervene were significantly more likely to express intentions to correct misinformation on social media. In the specific case of food health misinformation, this process may be even more salient: because misleading dietary claims can directly threaten everyday health decisions, activated personal norms strengthen individuals’ motivation to prevent harm by engaging in corrective behaviors.

**H4.**  *Personal norms regarding misinformation correction are positively associated with individuals’ intentions to engage in corrective behavioral outcomes.*

### 1.7. Multigroup Analysis by Media Literacy

Media literacy, conceptualized as an information processing competence that enables individuals to access, analyze, and critically evaluate media content, plays a pivotal role in how people respond to misinformation (Potter, 2004; Livingstone, 2004). Higher levels of media literacy equip individuals with stronger analytical skills, allowing them to detect misleading claims, assess credibility, and recognize potential risks associated with misinformation.

This competence can moderate key relationships in the proposed framework. Specifically, individuals with higher media literacy are more likely to connect presumed exposure in others to misinformation with perceptions of its harmful influence, and more likely to translate perceived harm into a sense of personal responsibility and normative obligation. By contrast, those with lower media literacy may fail to recognize misinformation as problematic, weakening the cognitive and normative links that precede corrective intentions.

Food health misinformation provides a particularly relevant context for examining this moderating effect. Because such content often appears as benign wellness tips or traditional wisdom, individuals with limited media literacy may not perceive its risks, whereas those with higher literacy can critically deconstruct these claims and more strongly infer their harmful consequences.

**Research Question (RQ):** How do the relationships among individuals’ attention to food health misinformation, perceptions of others’ exposure, perceived negative influence, personal norms, and corrective behaviors differ among participants with different levels of media literacy?

## 2. Materials and Methods

### 2.1. Data Collection

The sampling strategy used convenience sampling to recruit 1021 Chinese citizens in March 2025 via the Credamo platform, a professional data-collection service in China that provides data solutions to researchers from over 3,000 universities globally, comparable to platforms such as Qualtrics and Amazon Mechanical Turk (MTurk) (Z. Chen et al., 2024). Participation was voluntary, free of charge, and based on informed consent. The recruited participants exhibited a diverse demographic profile. Participants were eligible to receive a monetary reward of ¥3 CNY (US$0.42), which was credited directly to their electronic accounts. Following initial screening, individuals who completed the questionnaire in less than 3 min or provided identical responses to all questions were excluded from the analysis. The final sample consisted of 1021 participants, with 59.8% being women (*n* = 611). The age of participants ranged from 18 to 65 years (M = 31.67, SD = 6.96). In terms of monthly income, the majority of participants (*n* = 601, 58.9%) reported earnings ranging from ¥5000 to ¥14,999. Additionally, the sample was highly educated, with 91.7% (*n* = 936) of respondents having attained a higher education level. Specifically, 70.3% (*n* = 718) held a bachelor’s degree, while 21.4% (*n* = 218) possessed advanced degrees (MA or PhD).

### 2.2. Measures

Media attention to food health misinformation was focused on participants’ attention to social media. In previous research, scholars adopted just one single item to measure attention toward the issue on the Internet (Ho et al., 2015, 2020; Liao et al., 2016). Here, we adapted one item from Ho et al. (2015) with a seven-point Likert scale. How much attention (1 = no attention at all and 7 = very close attention) you paid on social media about food health misinformation (M = 4.64, SD = 1.07) and how much attention others paid on social media about food health misinformation (M = 4.75, SD = 1.03).

Perceived negative influence on others will be measured using a 7-point scale (1 = *strongly disagree*, 7 = *strongly agree*) adapted from Wan et al. (2003). Respondents will be asked to what extent they agree with in general (1) Food health misinformation has a substantial impact on others; (2) Food health of recognized brand misinformation has a substantial impact on others; (3) Food health of unrecognized brand misinformation has a substantial impact on others (M = 5.69, SD = 0.61).

Personal Norms will be measured by four items, using a 7-point scale (1 = *strongly disagree*, 7 = *strongly agree*) adapted from Bamberg et al. (2007) and Onwezen et al. (2013). For example, (1) I feel it is my moral obligation to correct food health misinformation (2) I feel that I should correct food health misinformation (3) I feel it is important that people in general correct food health misinformation (4) Regardless of what other people do, I feel an obligation to correct food health misinformation (M = 5.33, SD = 0.86, Cronbach’s α = 0.85).

Corrective behavioural outcomes will be measured using four items on a 7-point Likert-type scale (1 = *very unlikely*, 7 = *very likely*) adapted from L. Chen and Fu (2022) and Lim (2017). The respondents will be asked to indicate how likely they are to (1) sharing corrective information to relatives and friends through social media platforms (2) leaving a debunking comment under the misinformation, (3) posting debunking information under one’s social media account and (4) reporting misinformation to regulatory agencies to warn of potential risks (M = 3.92, SD = 1.23, Cronbach’s α = 0.86)

Media literacy will be measured using four items on a 7-point scale (1 = *strongly disagree*, 7 = *strongly agree*) adapted from Nienierza et al. (2021), which encompasses the dimensions of factual competence, self-competence, and social competence (Obermaier & Schmuck, 2022). Respondents will be asked to what extent they agree the following statements: (1) I always find the information I am looking for online (2) I find it easy to assess the accuracy of content on the Internet (3) I am well informed about how to protect my privacy on social media (4) I think about what I post on the Internet and what impact it could have on others (M = 5.38, SD = 0.67, Cronbach’s α = 0.63).

## 3. Result

### 3.1. Structural Equation Modeling for IPMI Model

To assess potential common method bias, we conducted Harman’s single-factor test using a CFA approach by loading all measurement items onto one latent factor. The one-factor model showed poor fit: $\chi^{2}(94)=1459.50,\;p<0.001$, RMSEA = 0.119 (90% CI [0.114, 0.125]), CFI = 0.744, TLI = 0.714, and SRMR = 0.099. These results indicate that a single factor does not adequately account for the covariance among the measures, suggesting that common method bias is unlikely to be a serious concern.

To address our hypotheses, we conducted structural equation modeling using a conventional two-step procedure. First, we evaluated the measurement model using CFA to confirm the adequacy of the latent constructs (i.e., factor loadings and overall model fit). Second, after establishing satisfactory measurement properties, we estimated the structural model by specifying the hypothesized paths among the latent and observed variables to test the proposed direct and indirect (serial mediation) effects.

First, we assessed the factor loadings of each latent variable and the model fit of the measurement model. The results demonstrated good factor loadings with all factor loadings exceeding 0.60. In addition, the measurement model revealed an excellent model fit (χ^2^ = 142.890, df = 41, *p* < 0.00; χ^2^/df = 3.49; CFI = 0.98; TLI = 0.97; RMSEA = 0.049; SRMR = 0.056). Based on the measurement model, we conducted a structural equation model by linking latent variables and observed variables. The structural model fit indices indicated an acceptable level (χ^2^ = 229.457, df = 78, *p* < 0.001; χ^2^/df = 2.94; CFI = 0.97; TLI = 0.96; RMSEA = 0.044; SRMR = 0.052) (L. Chen & Fu, 2022).

The structural model showed that media attention on self was positively associated with presumed exposure on others (β=0.623, SE = 0.019, p<0.001). Presumed exposure on others was, in turn, positively associated with presumed negative influence on others (β=0.280, SE = 0.039, p<0.001). Higher presumed negative influence on others was associated with stronger personal norms (β=0.331, SE = 0.040, p<0.001), and stronger personal norms were associated with more corrective behaviors (β=0.759, SE = 0.019, p<0.001). Finally, even after accounting for the mediated pathways, media attention on self still had a significant positive direct association with corrective behaviors (β=0.065, SE = 0.025, p=0.009).

Decomposition of effects indicated a significant total effect of Media attention on self on Corrective behaviors (Estimate = 0.079, SE = 0.019, p<0.001). The direct effect was also significant (Estimate = 0.047, SE = 0.018, p=0.009). Importantly, the total indirect effect was significant based on bootstrapping (Estimate = 0.032, SE = 0.007, p<0.001). The significant indirect effect was accounted for by the serial mediation pathway from media attention on self to corrective behaviors via presumed exposure on others, presumed negative influence on others, and personal norms (specific indirect = 0.032, SE = 0.007, p<0.001), supporting the proposed sequential mechanism.

### 3.2. Multi-Group Analysis

**Figure 1:**
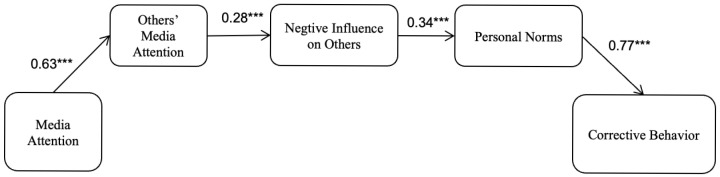
The overall influence of the presumed media influence model. N = 1021. Age and gender were introduced to the model as control variable. *** *p* < 0.001.

**Figure 2:**
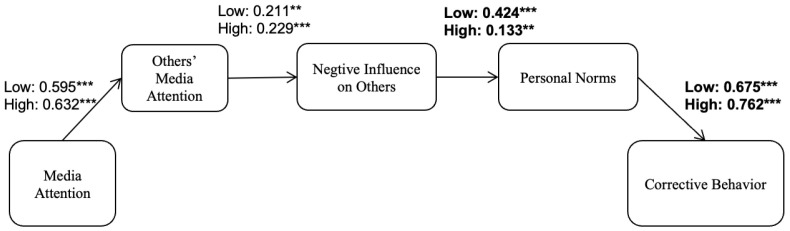
Multigroup analyses for low versus high-media-literacy groups. The path coefficients in bold represent significantly different coefficients. Age and gender were introduced to the model as a control variable. *** *p* < 0.001, ** *p* < 0.01.

Due to space constraints, the direct effects are directly reflected in the model plots (See Figure 1). A multi-group analysis was conducted to explore whether the model differed between participants with high versus low levels of media literacy. The analysis found that the structural model significantly differed between the two groups, Δχ^2^(1) = 56.13, *p* < 0.001 (see Table 1). Figure 2 visually displays the results. Subsequently, a series of chi-square difference tests were performed to compare various single-path constrained models against the unconstrained model.

## 4. Discussion

This study extends the Influence of Presumed Media Influence (IPMI) model to the context of food health misinformation and validates a sequential pathway from media attention to corrective behavioral intentions. Findings show that individuals who paid more attention to food health misinformation were more likely to presume that others had been exposed to such content, perceive it as harmful to others, develop stronger personal norms, and ultimately report greater willingness to engage in corrective behaviors. These results demonstrate that IPMI serves as a robust explanatory framework for understanding how users move from concern for others to prosocial action in digital environments (Gunther & Storey, 2003; Ho et al., 2015). Importantly, this study highlights the role of media literacy through multi-group analysis. The results indicate that the IPMI process does not operate uniformly across individuals but varies depending on levels of media literacy. This subgroup analysis is not only a methodological contribution but also a theoretical advancement, showing that the mechanisms of IPMI depend on individuals’ processing capacity. In other words, whether users can transform perceived social risks into personal norms and corrective action is contingent on their media literacy, echoing prior work on heterogeneity in misinformation processing (Vraga & Tully, 2021).

The results provide empirical confirmation and theoretical elaboration of the IPMI model in the context of food health misinformation. Building on the third-person effect (Perloff, 1999), IPMI proposes that perceptions of media influence on others can trigger a cascade of psychological responses, including personal normative beliefs and behavioral intentions. The findings of this study support this proposition, demonstrating that greater attention to food health misinformation anchors individuals’ assumptions that others have also been exposed. This extends earlier findings that attention functions as a perceptual anchor shaping inferences about broader audience exposure (Tsfati & Cappella, 2005; R. Wang & Huang, 2020).

The path from presumed exposure to perceived negative influence further clarifies the motivational structure of IPMI. Individuals not only believe that others have encountered misinformation but also evaluate that exposure as consequential and potentially harmful. In the context of food and health misinformation, concern arises less from factual inaccuracies alone than from the imagined behavioral consequences misinformation may trigger. Presumed influence thus functions as a signal of social risk, which in turn activates personal norms, consistent with the Norm Activation Model’s emphasis on awareness of consequences and ascription of responsibility (Schwartz, 1977; Bamberg & Möser, 2007).

This activation of personal norms predicts a sense of responsibility to act, particularly in contexts where institutional correction is lacking or delayed. These results suggest that IPMI is not only a perceptual framework but also a model of how external risk perceptions can be internalized into normative obligations within participatory media environments. Prior studies on corrective action in social media similarly highlight the importance of normative responsibility in motivating user-driven interventions (Bode & Vraga, 2015; Margolin et al., 2018).

The path from personal norms to corrective behaviors further demonstrates the explanatory power of IPMI for user-initiated intervention. Individuals are not passive recipients or evaluators of media content but become active agents who take responsibility for mitigating informational harm. This extends IPMI beyond traditional applications such as opinion shifts or regulatory support, underscoring its relevance for understanding bottom-up prosocial behavior in digital environments (Tsfati & Cohen, 2005).

Finally, our multigroup analysis deepens understanding of whether and how the IPMI process operates differently for people with higher versus lower media literacy. Overall, the sequential process linking attention to food/health misinformation to corrective behaviors through presumed exposure, perceived negative influence on others, and personal norms was supported in both groups; however, two pathways differed significantly in strength. Among individuals with lower media literacy, the association between perceived negative influence (harm) and personal norms was stronger, suggesting that those with more limited processing capacity may rely more on affective threat appraisals when forming normative commitments. In contrast, the link from personal norms to corrective behaviors was stronger among individuals with higher media literacy, indicating that although their normative commitments may be less affectively triggered, they are better able to translate these commitments into concrete actions. Taken together, these findings show that the IPMI mechanism is differentiated and layered, with the role of personal norms in misinformation correction contingent on individuals’ evaluative and behavioral capacities (Lewandowsky et al., 2017; Vraga & Tully, 2021).

### 4.1. Theoretical and Practical Implications

Theoretically, this study refines the IPMI framework by showing that concern for others can drive not only attitudinal change but also norm-based behavioral intentions. By modeling the sequential pathway from media attention to presumed influence, perceived harm, personal norms, and corrective behavior, it clarifies the psychological structure through which users process and act upon misinformation. This extends IPMI beyond a perceptual model, positioning it as a framework that explains how perceptions of social risk are internalized into normative commitments and behavioral engagement.

A distinctive contribution lies in identifying media literacy as a key boundary condition. The moderation analysis reveals that IPMI processes are not uniform across individuals: lower-literacy users may rely more on affective reactions when forming personal norms, while higher-literacy users are better able to translate those norms into deliberate corrective actions. This highlights audience heterogeneity and demonstrates that media literacy is not just a background skill but a theoretical mechanism that determines how social concern is converted into behavioral engagement.

Practically, these findings suggest that interventions against misinformation should move beyond factual correction or exposure reduction. Communication strategies that emphasize shared social risks and personal responsibility may better mobilize grassroots corrective actions. At the same time, media literacy should be treated as an enabling capacity for proactive engagement. Initiatives that combine cognitive evaluation with normative reflection can empower users to critically assess content and act on their personal responsibility, thereby fostering more resilient information ecosystems.

### 4.2. Limitations and Directions for Future Research

This study has several limitations that should be acknowledged. First, the cross-sectional design restricts our ability to draw strong causal inferences about the sequential relationships specified in the IPMI framework. Although the proposed model is theoretically grounded and supported by the current evidence, future research using longitudinal or experimental designs would allow for a more rigorous validation of the temporal order and directionality of the pathways.

Second, the data were drawn from a single national sample in China. While the Chinese media context—with its strong emphasis on food safety—offers a meaningful setting for this study, media environments, cultural values, and civic norms vary across societies. Future studies should examine whether the IPMI mechanisms generalize to other cultural and political contexts, or whether they operate differently depending on media trust and regulation.

Third, the operationalization of “others” in this study was intentionally broad, without distinguishing among family members, friends, or broader publics. Different social referents may elicit varying levels of concern and responsibility, potentially shaping the activation of personal norms and corrective intentions. Future work could disaggregate “others” to clarify how relational closeness moderates the correction pathway.

Fourth, although this study introduced personal norms and media literacy as key extensions of the IPMI model, other psychological and motivational factors such as emotional arousal, perceived self-efficacy, or social desirability bias were not examined. Incorporating these variables in future research could provide a more comprehensive understanding of how individuals process misinformation and decide to intervene.

Finally, the external validity of this study should be interpreted with some caution. The model was tested in the specific context of food/health misinformation in China, where cultural norms, platform governance, and public sensitivity to food safety may differ from other countries, potentially affecting the strength of the IPMI pathways. Finally, we measured self-reported corrective intentions rather than observed behaviors, so generalizing to actual corrective actions across platforms and contexts should be done carefully.

### 4.3. Conclusions

This study extends the Influence of Presumed Media Influence (IPMI) model to food- and health-related misinformation and demonstrates a sequential pathway linking media attention to corrective behavioral intentions. Using a national online survey of Chinese adults, the SEM results support a serial process in which greater attention is associated with higher presumed exposure of others, stronger perceptions of negative influence on others, heightened personal norms, and, in turn, stronger intentions to correct misinformation. Importantly, the multi-group analysis indicates that this process varies by media literacy: the path from perceived negative influence to personal norms is stronger among participants with lower media literacy, whereas the path from personal norms to corrective intentions is stronger among those with higher media literacy.

## Figures and Tables

**Table 1:** Multigroup Comparisons of IPMI Model across Low- and High-Media-Literacy Groups (*N* = 1021).

Multigroup Models	χ^2^	*df*	χ^2^/*df*	CFI	TLI	RMSEA
Unconstrained	380.05	172	2.21	0.95	0.95	0.05
Constrained (All paths)	436.181	176	2.48	0.94	0.93	0.05
Chi-square difference test	Δχ^2^ (4) = 56.13, *p* < 0.001					
Constrained (Media Attention on Self → Media Attention on Others)	380.297	173	2.20	0.95	0.95	0.05
Chi-square difference test	Δχ^2^ (1) = 0.25, *p* = 0.62					
Constrained (Media Attention on Others → Negative Influence on Others)	381.238	173	2.20	0.95	0.95	0.05
Chi-square difference test	Δχ^2^ (1) = 1.19, *p* = 0.28					
Constrained (Negative Influence on Others → Personal Norms)	401.521	173	2.32	0.95	0.94	0.05
Chi-square difference test	Δχ^2^ (1) = 21.47, *p* < 0.001					
Constrained (Personal Norms → Corrective Behavior)	414.599	173	2.40	0.95	0.94	0.05
Chi-square difference test	Δχ^2^ (1) = 34.549, *p* < 0.001					

## Data Availability

Data can be available from corresponding author upon reasonable request.
